# eHealth usage among parents to premature or surgically treated neonates: associations with eHealth literacy, healthcare satisfaction or satisfaction with an eHealth device

**DOI:** 10.1186/s12887-023-04340-3

**Published:** 2023-10-21

**Authors:** Mariette Derwig, Rose-Marie Lindkvist, Inger Kristensson Hallström, Björn A. Johnsson, Pernilla Stenström

**Affiliations:** 1 Department of Health Sciences, Faculty of Medicine, Lund University, P.O. Box 157, Lund, SE-22100 Sweden; 2 Department of Computer Science, Lund University, Lund, Sweden; 3 Department of Paediatrics, Clinical Sciences Lund, Lund University, Skane University Hospital, Lund, Sweden

**Keywords:** eHealth, eHealth literacy, eHealth usage, Health care satisfaction, User satisfaction

## Abstract

**Background:**

A specific eHealth device, a surf tablet, was developed for bridging between advanced in-hospital care and children’s homes. Since little is known about determinators for parental eHealth usage, the study’s aim was to explore if parents’ usage of the device was associated with their eHealth literacy, or their satisfaction with their child’s healthcare or with the specific surf tablet.

**Methods:**

In this explorative usage and questionnaire study, parents to neonates who were discharged home after advanced in-hospital care were included. Their surf tablet usage at maximum 30 days after discharge was reported as frequency (%) of active days (usage days/days having the device) and median number of tablet activities (chat and photo) per usage day. eHealth literacy (eHealth Literacy Questionnaire; eHLQ), healthcare satisfaction (PedsQL Healthcare Satisfaction Generic Module), and satisfaction with the surf tablet were explored regarding tablet usage. Statistics were described in median (range) and (%) using non-parametric and regression models (p < 0.05).

**Results:**

Parents to 32 children (11 premature, 21 operated) were included. Active days with eHealth communication using the device was 39% (9.0/29.5), with 2.0 (1.0-4.2) usage occasions per active day. Activity on the tablet was higher among parents reporting to be very satisfied or satisfied with the device (n = 25) compared with neutral/dissatisfied parents (n = 7) (2.8 vs. 2.2 vs. 1.6 activities) (p = 0.030), while their frequency of active days did not differ (31.6% vs. 38.3% vs. 40%) (p = 0.963). A higher eHealth literacy was not associated with frequency of active days (0.926 (0.652–1.317); p = 0.659) or number of eHealth activities (0.973 (0.758–1.250); p = 0.825). Healthcare satisfaction was not associated with higher frequency of active days 0.996 (0.983–1.009; p = 0.519); neither was number of eHealth activities 1.001 (0.991–1.011; p = 0.883).

**Conclusion:**

In this study, eHealth usage was associated with parental satisfaction with the specific eHealth device, but not with eHealth literacy or healthcare satisfaction. To assure equal access to healthcare when using eHealth, the user-friendliness of the device seems to be crucial, and technical support needs to be in place.

**ClinicalTrials.gov registration identifier:**

NCT04150120 (04/11/2019).

**Supplementary Information:**

The online version contains supplementary material available at 10.1186/s12887-023-04340-3.

## Introduction

Digital technology is reported to be increasingly used for children with medically and surgically complex illnesses in paediatric healthcare [1]. The core idea of the eHealth solution presented here provides parents to children who have been treated at highly specialised paediatric hospital departments with direct digital communication to the specialised professionals during their child’s transfer period to home. This is, as reported, to enhance access to healthcare, improve family engagement, and facilitate parents’ interaction and communication with healthcare providers [2–4]. It has been suggested that modern eHealth solutions strengthen parents in their parental role, reduce parental stress, and decrease the number of emergency hospital visits [2, 5–7]. Parents using eHealth in their contact with neonatal units considered them both to be secure and easy to use [8, 9]. Yet, meeting parents’ and children’s individual needs in medical care, especially with regard to eHealth, has been highlighted as pivotal [10]. According to a review, one potential barrier to achieving successful eHealth usage is users’ lack of technological literacy [11]. Similarly, for effective interaction with eHealth services, a certain level of eHealth literacy is reported to be crucial [3, 7, 12, 13]. In our previous studies, parents provided with eHealth after discharge from hospital reported an increased access to healthcare, but also raised concerns about their own literacy [4, 5]. For eHealth in advanced paediatric care, knowledge about the association between eHealth usage and eHealth literacy is yet unknown, even though such knowledge would be of importance to guaranteeing equal access to healthcare.

Clear communication between parents and medical staff is reported to improve parental satisfaction with their child’s medical care [14], but there is a gap in knowledge about eHealth’s impact on the clarity of communication and if parental e-health literacy impacts parental usage of e-health. This could be of importance for getting insights in which parents who actually will use the eHealth device, and to prepare families accordingly. Therefore, the main aim of this study was to explore if parents’ usage of eHealth during transfer from hospital to home was associated with their eHealth literacy and their overall satisfaction with their child’s care related to prematurity or surgery for congenital colorectal malformations.

One factor reported to increase parents’ inclination to use eHealth is their satisfaction with the specific digital health device and the feasibility of the technology, e.g., its user-friendliness [3, 13]. Therefore, before implementing eHealth in advanced paediatric care, it is crucial to understand whether or not the application or device serves parents’ requirements, and if higher satisfaction with the device increases parents’ eHealth usage. This rationalises the secondary aim of the study; to describe the association between parents’ usage of eHealth and satisfaction with the eHealth device.

The hypothesis, according to previous reports, was that parents using the eHealth device most frequently were those with a higher eHealth literacy. The hypothesis was also that parents who reported higher satisfaction with their child’s healthcare would be those who used the eHealth device most frequently. These parents were considered to be more willing to continue their communication with the highly specialised paediatric care using the eHealth device in a home setting. For the same reason, parents who reported a high satisfaction with the eHealth device were hypothesised to be frequent users since they enjoyed the process.

## Materials and methods

### Design

This was an explorative study performed within the frame of a Swedish experimental controlled clinical trial developing and evaluating eHealth solutions for families after hospital care (ClinicalTrials.gov identifier: NCT04150120: 04/11/2019).

### Setting

The study was conducted at a tertiary neonatal department and a tertiary paediatric surgery department assigned as a national centre for anorectal malformations, Hirschsprung’s disease, congenital diaphragm hernia and oesophageal atresia. The catchment area covered 5 million residents, with travel distances of up to 1,000 km.

### Study population

Parents to prematurely born children and to children having gone through paediatric surgery for congenital malformations, leaving hospital for home, and able to read and write Swedish, were included between August 2019 and January 2022. Before leaving hospital, parents received written instructions and oral information about the eHealth device, and its multiple functionalities designed to facilitate communication with healthcare professionals. They also tested using the device before discharge to home. Inclusion criteria for the study were available eHealth usage data and completed questionnaires about eHealth literacy and healthcare satisfaction. For drop-out analyses, usage data of parents who did not answer the questionnaires at all, or fully, were used.

### The eHealth device

The eHealth device was developed in the form of an eHealth application installed on a surf tablet. The development period had taken place between 2016 and 2018 [15] using a participatory design, continuously collecting feedback from the users, including parents of premature children or those with surgically complex illnesses, and health professionals, as well as from representatives of patient organisations [15, 16]. Practically, when being discharged from hospital, the families were offered a surf tablet with an applied application to take home. Safe communication over the application was secured by a double encryption of the data. The application had a set of universally useful features, including video counselling (calls), direct bilateral messaging (chat), only between patients at home and healthcare professionals in hospital, and the facility for patients’ guardians to send photos. These were photos taken with the tablet’s camera for a secure transfer, storage, and review by the healthcare professionals. Furthermore, the double encrypted application was configured to meet the needs of specific patient groups, such as adding standardized diagnose-specific reports including questionnaires assessing weight, eating habits, bowel function etc. [17]. Use of the surf tablet was added onto the conventional care, which for neonates included regular home visits by a mobile home care team, using mobile phones for text messages, and planned physical counselling at the hospital. In paediatric surgery, the conventional post-operative care included landline hospital telephone to appointed specialist nurses and planned physical counselling at the hospital.

### Data collection

eHealth usage data was automatically transferred from the surf tablet to a central server. The eHealth usage of the tablet was assessed during a maximum period of 30 days after discharge from hospital. For this study’s aim, three out of the project’s 11 questionnaires were selected: The validated eHealth Literacy Questionnaire (eHLQ) [13], the Paediatric Quality of Life (PedsQL) Healthcare Satisfaction Generic Module [18], and the eHealth Satisfaction Questionnaire, which was a within-project designed questionnaire about parents’ satisfaction with communication over this specific eHealth device. Parents included were requested to answer eHealth related questionnaires online. Either one or both parents per child (or twins) answered the questionnaires. Each questionnaire was personal, i.e., had its own separate link.

#### Background and pertinent variables

Information on parental socioeconomic and demographic factors such as age, education (primary, secondary, university levels), distance from home to hospital (kilometres) as well as the child’s diagnosis, were collected from a general background questionnaire.

#### eHealth usage analysis

Statistics on usage of the eHealth device, including individual messages, images and standardised reports, was compiled from a central secure storage server. Usage effective time was set as having the eHealth device at a maximum of 30 days from hospital discharge, including both start and end date. If the period of having the eHealth device at home was shorter than 30 days, the exact end date was used. The output parameters assessed over the time of having the eHealth device were: (1) Total number of days having the eHealth device (effective days), and (2) Number of active usage days, which was the number of days when the eHealth device was used for at least one activity of any kind. The parameters, calculated from the output data, were: (1) Ratio of active days (%) corresponding to the number of active days divided by the number of days having the surf tablet (effective days within a maximum 30 days), and (2) Average number of activities corresponding to the average number of activities (n) on active days, i.e., total number of activities divided by the number of active days. These parameters were used for both the main and drop-out analysis.

#### eHealth literacy

The self-reported eHealth Literacy Questionnaire (eHLQ) [13] evaluates people’s self-perceived interaction with digital health services and technology in relation to their health. It contains 35 items describing parents’ individual competence in how technology can promote their own health (domains 1 to 3), the interaction between the parent and the digital services (domains 4 and 5) and parents’ experiences with digital health services for their own health (domains 6 and 7). Each item is scored using a four graded ordinal scale: 1 (strongly disagree), 2 (disagree), 3 (agree) and 4 (strongly agree). The highest score signifies the highest eHealth literacy, while the lowest scale score indicates the lowest eHealth literacy [19]. Two of the domains were selected for this study since they could potentially be associated with eHealth usage: domain eHLQ 3: Ability to actively engage with digital services with the following questions: ‘*I know how to get information using technology…; I know how to make health technology work…; I can enter data…; I quickly learn how to…; I easily learn to use…*’ and domain eHLQ 5: Motivation to engage with digital technology used: ‘*Technology makes me feel actively…; I find technology helps me…; I find I get better care…; Technology improves my communication…; I find technology useful*’.

#### Healthcare satisfaction

Healthcare satisfaction was assessed by dimension 6 of the Paediatric Quality of Life Score (PedsQL) Healthcare Satisfaction Generic Module [18, 20]. PedsQL comprises 24 items concerning six dimensions: (1) Information (five items); (2) Inclusion of Family (four items); (3) Communication (five items); (4) Technical Skills (three items); (5) Emotional Needs (four items); and lastly (6) Overall Satisfaction (three items), which was the dimension selected for this study. Answers were given on a five-point Likert scale: 0 (Never), 1 (Sometimes), 2 (Often), 3 (Almost always) to 4 (Always) transformed to a 0-100 scale according to; 0 = 0, 1 = 25, 2 = 50, 3 = 75, 4 = 100. Higher scores indicated higher satisfaction. Dimension 6, covering overall healthcare satisfaction, comprised the following three questions: *‘I am ……satisfied about 1) the overall care my child is receiving, 2) how friendly and helpful the staff is, and 3) the way my child is treated at the hospital.’*

#### eHealth device satisfaction

Parental satisfaction with the eHealth device was assessed by the selected question: “How satisfied were you to communicate from home with healthcare professionals, via the eHealth device?” This question constituted a part of the evaluation of the whole eHealth study setting. Answering options were on a five-point Likert scale ranging from ‘very dissatisfied’ to ‘very satisfied’, with ‘neither dissatisfied nor satisfied’ in the middle. Parental satisfaction with the eHealth device was, in accordance with literacy and healthcare satisfaction, analysed regarding eHealth device usage.

### Statistical analysis

Usage data were measured per child. Therefore, if two parents of the same child had answered the questionnaires, to associate their usage with questionnaire answers, their answers were combined into one observation. The combined answers were calculated using a mean of the two parents’ ages and a mean of their scores regarding eHealth literacy and healthcare satisfaction, respectively. For the satisfaction with the eHealth device, the lowest grade answer of any of the parents was selected for the analysis. The educational level was dichotomised into if at least one of the parents had received university education.

Background information and usage were given in descriptive data, both separate for neonatal and paediatric surgery, as well as in a total. The total was used for all statistics. Linear regressions, using unadjusted and adjusted models, were used for statistical analyses for association between eHealth usage and eHealth literacy, or for healthcare satisfaction. To meet the assumptions of linear regression, the outcome variables were log-transformed. Results for both the unadjusted and adjusted model (controlling for age and university education) were presented with estimate, 95% confidence interval, and p-value.

Analyses of eHealth device satisfaction was analysed with regard to three levels of satisfaction: Very satisfied, satisfied, and neutral/dissatisfied. Parents who answered ‘very dissatisfied’, ‘dissatisfied’ and ‘neither dissatisfied nor satisfied’ were compiled to one group ‘dissatisfied/ neutral’ to avoid revealing any single participant, as the numbers in ‘very dissatisfied’ and ‘dissatisfied’ were low. Usage of the device was analysed against satisfaction with the device, by using Kruskal-Wallis tests. If any difference was identified, pairwise Mann-Whitney U-tests, adjusted with the Bonferroni correction for multiple comparisons, were used.

## Results

**Fig. 1:**
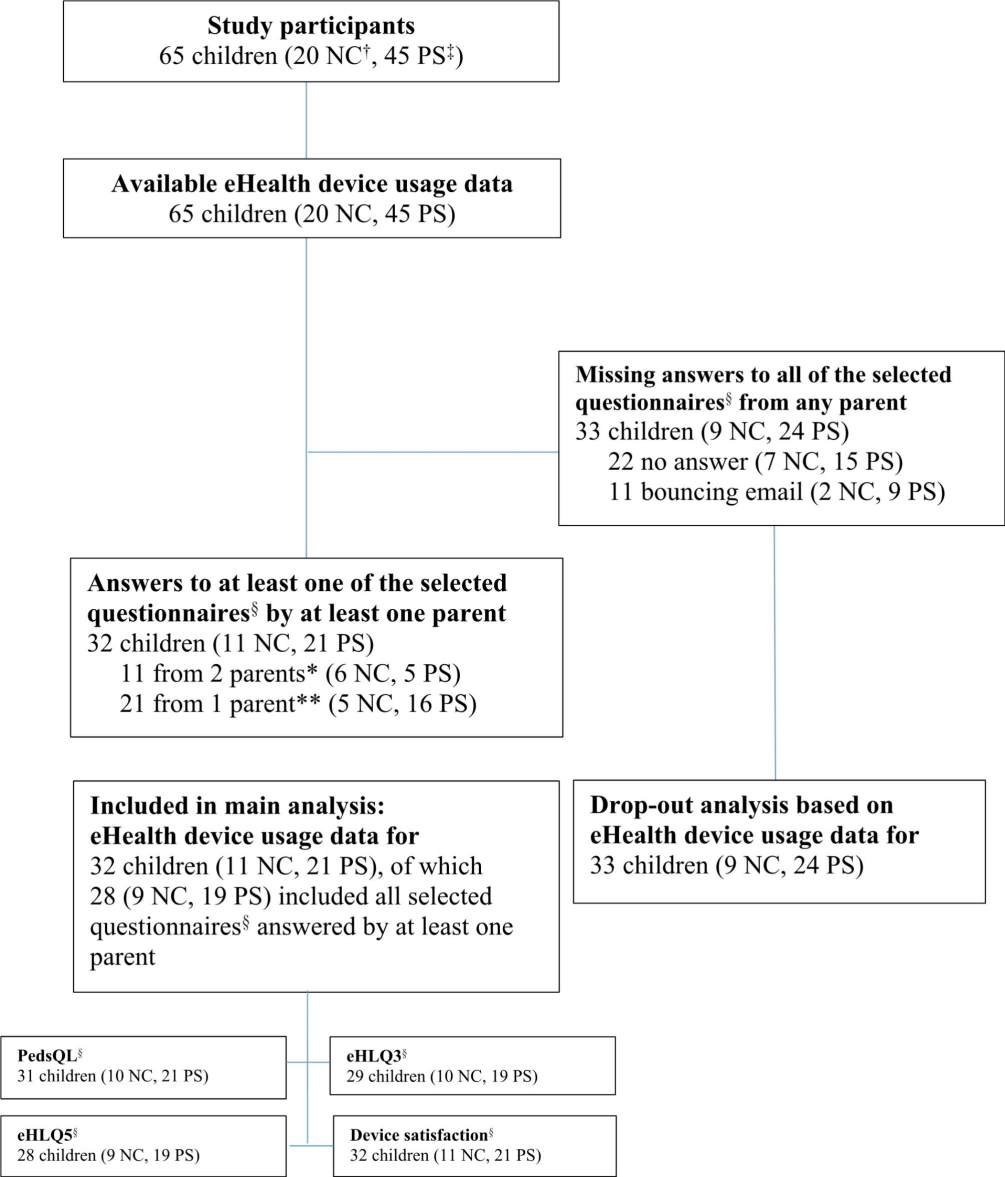
Diagram of participant inclusion flowchart

†NC = Neonatal care. ‡PC = Paediatric surgery care. § The following questionnaires were selected: PedsQL Healthcare Satisfaction Generic Module [17, 19]; eHealth Literacy Questionnaire (eHLQ) [13] and Device satisfaction: a specifically within-project designed questionnaire about parents’ satisfaction with communication using this specific eHealth device. *11 mothers and fathers; **13 mothers and eight fathers.

Parents to a total of 65 children with prematurity (n = 20) and surgery for congenital malformations (n = 45) agreed to participate in the study. After exclusions of missing usage data or incompletely answered questionnaires, 45 parents to 32 children (11 neonatal care and 21 paediatric surgery) including two pair of twins, were included (Fig. 1).

### Background information

The parents had a median age of 34 years, 56% were women, and 75% had at least university education (Table 1). All parents were born in a European country, and no one lived in separate households. The families lived median 59 km from hospital (Table 1). The premature children were born in gestational week 28–32 and children in the paediatric surgery group (n = 21) were treated for anorectal malformations (n = 9), Hirschsprung’s disease (n = 6), oesophagus atresia (n = 3), gastrointestinal atresia (n = 2), and congenital diaphragm hernia (n = 1).

**Table 1:** Descriptive statistics of variables included in the study, neonatal care and paediatric surgery, respectively

	Neonatal (n = 11)	Surgery (n = 21)	Overall (n = 32)	Missing
	median [min, max]	median [min, max]	median [min, max]	n (%)
Age parents (years)	34.5 [27.0, 44.0]	34.0 [26.0, 55.0]	34.0 [26.0, 55.0]	0 (0.0%)
University education	8 (72.8%)	16 (76.2%)	24 (75.0%)	0 (0.0%)
Distance between hospital and home (km)*	16.8 (11.6–24.2)	248.0 (14–343)	58.9 (11.6–343)	0 (0.0%)
Ability to engage in eHealth 1-max 4	3.5 [3.0, 4.0]	3.4 [1.4, 4.0]	3.5 [1.4, 4.0]	3 (9.4%)
Motivation to engage in eHealth 1-max 4	3.0 [2.2, 3.7]	2.9 [2.2, 4.0]	3.0 [2.2, 4.0]	4 (12.5%)
Overall healthcare satisfaction 0-max 100	95.8 [75.0, 100]	100 [50.0, 100]	100 [50.0, 100]	1 (3.1%)
Total number of days with device at home	28.0 [14, 68]	36.0 [8, 299]	30.0 [8, 299]	0 (0.0%)
Number of days with device at home (max 30 days)	28.0 |14, 30]	30.0 [8, 30]	29.5 [8, 30]	0 (0.0%)
Percentage of active days/ days with device (max 30)	40.0 [14.3, 70.0]	38.9 [20.0, 86.7]	39.4 [14.3, 86.7]	0 (0.0%)
Average number of activities on active days	1.20 [1.00, 1.82]	2.5 [1.50, 4.20]	2.00 [1.00, 4.20]	0 (0.0%)
How satisfied were you with communication via eHealth device?				0 (0.0%)
Very dissatisfied	0 (0.0%)	0 (0.0%)	0 (0.0%)	
Dissatisfied	2 (18.2%)	0 (0.0%)	2 (6.3%)	
Neither dissatisfied nor satisfied	4 (36.4%)	1 (4.8%)	5 (15.6%)	
Satisfied	3 (27.3%)	8 (38.1%)	11 (34.4%)	
Very satisfied	2 (18.2%)	12 (57.1%)	14 (40.6%)	

*95% of children (20/21) within paediatric surgery were treated for diagnoses related to national specialised medical care.

The drop-out group, i.e., those who had not answered the questionnaires at all or fully, but whose data of usage of the device could be compiled, included parents to 33 children. The distribution of prematurity and surgically treated conditions did not differ significantly compared to the study group; children to parents in the drop-out group had been hospital treated due to prematurity (n = 9) in gestational weeks 28–32, and had paediatric surgery (n = 24) for anorectal malformations (n = 10), Hirschsprung’s disease (n = 6), oesophagus atresia (n = 4) and other gastrointestinal malformations (n = 4).

### Usage of the eHealth device

**Fig. 2:**
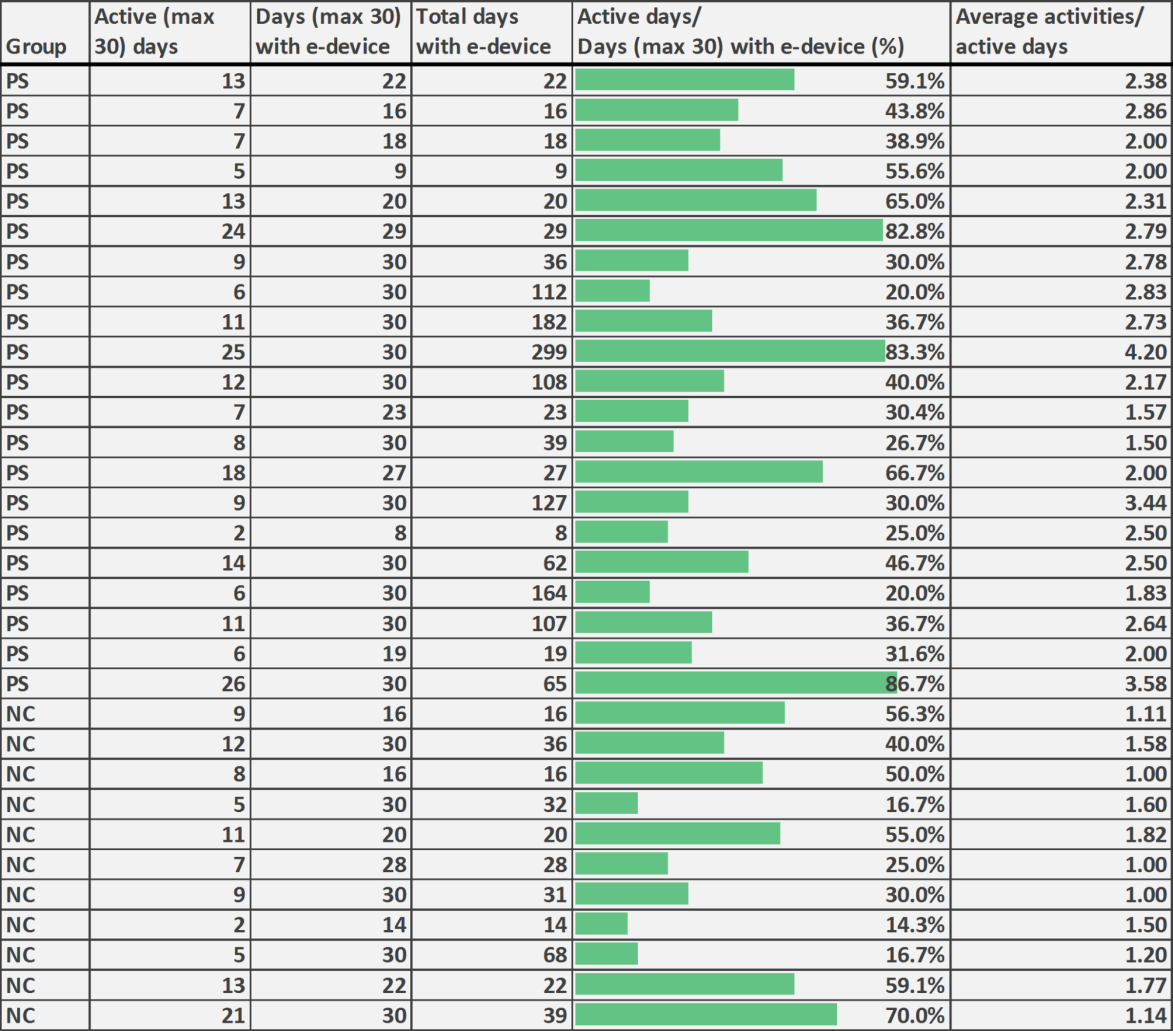
eHealth usage at the individual level. PC = Paediatric surgery care. NC = Neonatal care

The median time for having the surf tablet at home, maximum 30 days, was 29.5 days (Table 1). 50% returned the device before 30 days (16/32), while the other 50% (16/32) kept it longer (Fig. 2).

The percentage of active days of eHealth usage (active days/days having the eHealth device a maximum of 30 days) was median 39.4% (9.0/29.5). On each active day, parents sent median 2.0 activities in forms of messages, images, or standard reports (Table 1). In the drop-out group, the percentage of active usage days was median 26.7% (7.0/30.0) and median activities per active usage day was 2.2 (data not shown).

### eHealth literacy and eHealth usage

eHealth literacy, assessed both by ability to engage in eHealth and motivation to engage in eHealth (both with score 1–4; 4 = strongest literacy), was scored median 3.5 and 3.0, respectively (Table 1). A higher ability to engage in eHealth was not associated with a higher eHealth usage neither in frequency of active days: 0.926 (0.652–1.317), p = 0.659, or number of eHealth activities: 0.973 (0.758–1.250), p = 0.825 (Table 2). Neither was a higher score in motivation to engage in eHealth associated with more active days of eHealth usage: 1.149 (0.758–1.742), p = 0.489, or more eHealth activities per active day: 1.026 (0.766–1.375), p = 0.858 (Table 2). The absence of association between eHealth literacy and eHealth usage did not change after adjustments for parents’ age and education (data not shown).

**Table 2:** Estimated effects of eHealth literacy on eHealth usage and overall healthcare satisfaction

	Unadjusted models		Adjusted models	
	Exp (Beta) (95% CI)	*p-value*	Exp (Beta) (95% CI)	*p-value*
Percentage of active days/days with eHealth device for a maximum of 30 days (%)				
Ability to engage in eHealth	0.926 (0.652–1.317)	0.659	0.952 (0.658–1.375)	0.784
Motivation to engage in eHealth	1.149 (0.758–1.742)	0.498	1.141 (0.750–1.735)	0.522
Overall healthcare satisfaction	0.996 (0.982–1.01)	0.540	-0.997 (0.983–1.011)	0.676
Average activities on active days with the eHealth device				
Ability to engage in eHealth	0.973 (0.758–1.250)	0.825	0.963 (0.733–1.266)	0.781
Motivation to engage in eHealth	1.026 (0.766–1.375)	0.858	1.029 (0.750–1.413)	0.853
Overall healthcare satisfaction	1.001 (0.991–1.011)	0.833	1.001 (0.990–1.001)	0.898

### Healthcare satisfaction and eHealth usage

The overall satisfaction with healthcare (0-100; 100 = highest satisfaction) was scored median 100 (range 50–100) (Table 1). A higher score of healthcare satisfaction was not associated with a higher frequency of active days: 0.996 (0.983–1.009), p = 0.519, or more eHealth activities per active day: 1.001 (0.991–1.011), p = 0.883 (Supplemental 1a and b).

The absence of association did not change after adjustments for parents’ age and education (data not shown).

### Satisfaction with the eHealth device and eHealth usage

**Fig. 3:**
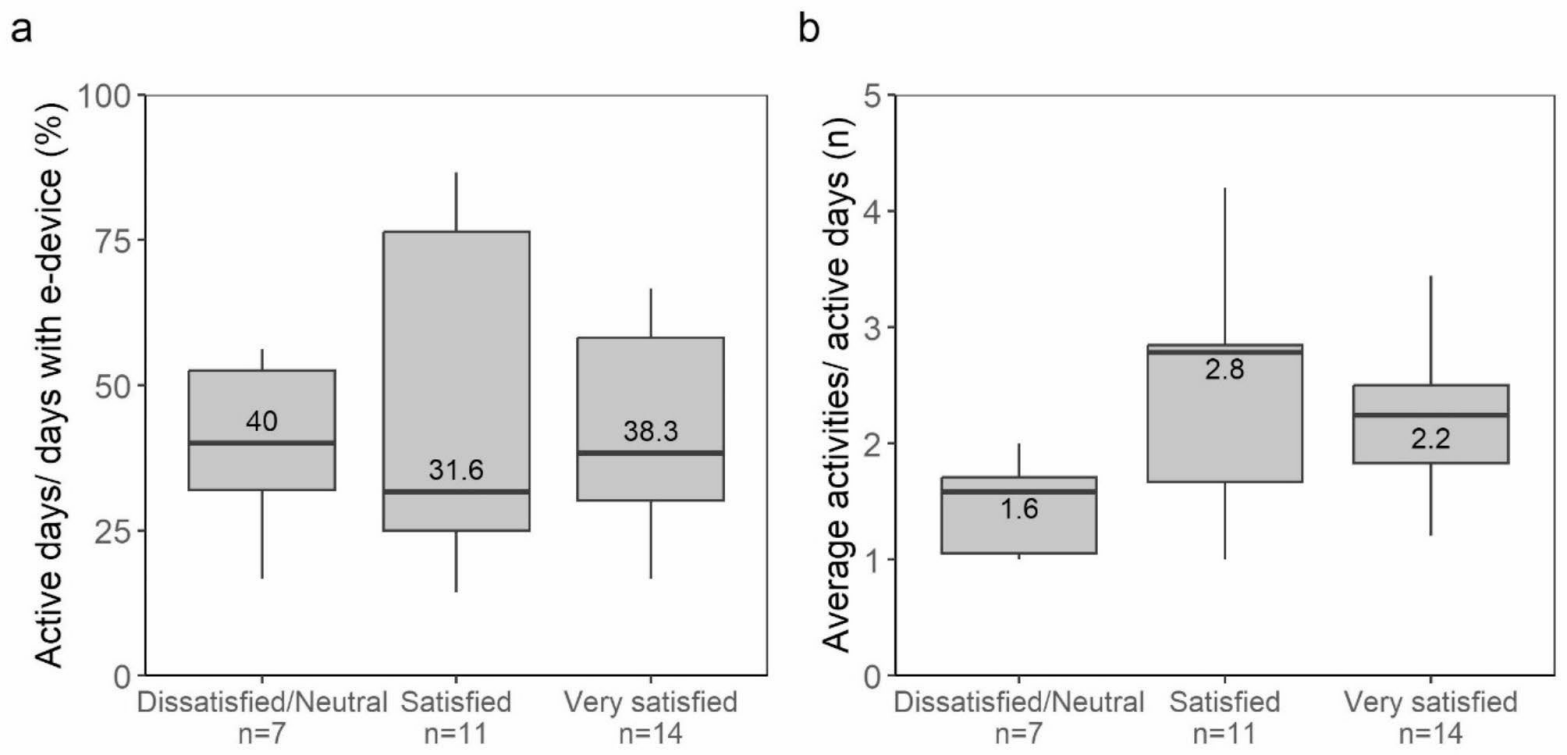
Boxplots visualising parents’ reported satisfaction with the eHealth device associated with its usage

In total, 40.6% (14/32) of parents to children (two neonatal and 12 paediatric surgery) answered that they were very satisfied with the specific eHealth device, and 34.9% (11/32) answered that they were satisfied. (Table 1). These satisfied and very satisfied parents (n = 25; 78%) used the eHealth device more frequently compared to parents reporting being neutral/dissatisfied with the device (n = 7; 22%) in terms of having more activities per active day (median 2.8 vs. 2.2 vs. 1.6) (p = 0.030) (Fig. 3). The frequency of active usage days did not differ between the satisfaction groups (31.6% vs. 38.3% vs. 40.0%) (p = 0.963). (Fig. 3).

The black line within the boxplots represents the median): (a) Active days (%) did not differ between parents reporting being dissatisfied (n = 5)/neutral (n = 2), satisfied n = 11, or very satisfied n = 14 with the eHealth device (p = 0.963); (b) Number of activities per active days were more frequent among parents reporting being satisfied and very satisfied (p = 0.030; Kruskal Wallis).

## Discussion

In this study, the usage of eHealth in the form of a surf tablet, in the transition to home after discharge from advanced paediatric surgery and prematurity in-hospital care, was associated with parental satisfaction with the eHealth device but not with eHealth literacy nor with healthcare satisfaction.

Our results showed that parents with a higher satisfaction of the eHealth device also used the device to a greater extent. This implies that the eHealth device’s user-friendliness impacted on its usage, and that the usability of the device, its interface and human factor engineering, played a central role. However, since this study did not explore any underlaying reasons for this association, our data cannot exclude the opposite association, i.e., that frequent use of the device resulted in a more positive experience and personal confidence with the surf tablet. Most probably, if speculating, the association was caused by bilateral triggering effects of usage and device satisfaction. The result is in line with some other research results indicating that personal experiences of eHealth can predict a degree of eHealth usage [21, 22]. Contrarily, absence of associations between satisfaction with eHealth services and eHealth usage have also been described [23]. Differences between results could be attributed to various target groups, settings, and types of eHealth interventions. Also adding to uncertainties is that there are few current available eHealth studies, which is why interpretation of results and their transfer to local settings and digital solutions can be challenging.

One important aspect that cannot be underestimated when introducing health information technologies is the human factor; i.e. the users’ needs, capabilities, and limitations. If these are not properly considered during the design process, the technologies may be abandoned outright [24]. Overall, in our setting and with the specific eHealth solution most parents reported a very high or high satisfaction with the device. This high rate of satisfied parents could be due to the foregoing development process of the eHealth device, using a participatory design methodology, including both end-users and healthcare professionals from the start [5, 15]. This participatory approach is known to allow a responsive dialogue with the end-users based on cooperative activity between users and the designers, specifically ending up in a contemplative and quick prototyping [25, 26]. Further, such proactive partnerships have been reported to produce meaningful innovations in eHealth interventions [27]. In line with this most parents in our study were satisfied or very satisfied with the eHealth device. Therefore, a participatory design could be recommended when setting up a development plan for eHealth introduction.

The parents’ highly experienced satisfaction with the device might also be attributable to the thorough information and instructions parents were given about the device before leaving the hospital. Parents were thoroughly informed about the technical details and reassured that communication over the device was enabled only through encrypted communication in both the end-to-end communication and in the database. This in-depth information and the efforts taken to train users on the surf tablet, as well as informing them about its security, has its background in our research project’s previous results. Parents specifically highlighted the importance of experiencing secure information transmittance within eHealth when using the surf tablet. They reported that a safe and protected way to handle their sensitive chats and images of their children was of great concern to them [4, 5]. In the same studies, parents described that the technical issues causing obstacles in communication also caused uncertainties regarding the technology, and doubtfulness regarding security of the transmitted data [4, 5, 15].

In our study, reasons for low parental satisfaction with the device were not explored. Although few parents reported being dissatisfied with the device (two from neonatal care), or neutral (five being neither dissatisfied nor satisfied), an understanding of the causes leading to low satisfaction with the device would be important. With such information, the design of eHealth solutions could be better adapted and improved. Speculating, one reason for lower satisfaction (in line with previously mentioned findings about the impact of technical obstacles) could be that the technical problems hindered regular contact with health professionals via the eHealth device. In summary, after analyses, the positive association between satisfaction with the eHealth device and parental usage of eHealth, suggests the importance of eHealth device design, usage information, and data security.

In our study, usage of eHealth was not associated with eHealth literacy. eHealth literacy with regard to usage has not been widely explored within the paediatrics field. However, contrary our results, one of the rare paediatric studies showed a relatively strong association between eHealth literacy and usage of eHealth resources among parents of children with complex congenital heart defects [27]. Also contrary our results, stand those from a study of elderly users in a senior welfare centre [28]. In our study, the scores of technical literacy were high, both with regard to ability and motivation to engage in eHealth (median scores 4.3/5). This could be due to the considerably lower age of participants, who are expected to be frequent users to digital technology in other areas, and to their high educational level. For unknown reasons, parents in our study had a higher education (75% had university level) than the general Swedish population aged 25–64, of which 44% in 2020 were reported as university educated [29]. The eHealth literacy scores and education levels in this study did not differ from the scores in a historical control group within the same project, including parents not receiving the eHealth device after discharge from hospital following paediatric surgery [18]. Reasons for the high education level, and therefore a possible selection bias, can only be speculated on.

Even more pronounced than the high health literacy scores were the scores of healthcare satisfaction, where the median score was 100 (the maximum). Such skewness might influence statistical outcome. In another Swedish study that used the PedsQL Healthcare Satisfaction for assessing parental satisfaction with homecare services for their child, parents reported a high PedsQL satisfaction score (mean 84.5 (± 11.8)) [30]. Speculating, reasons for the high satisfaction in our study could be that highly specialised medical healthcare could easily be attributed high-quality care, due to the unique competence associated it. Rare diseases such as malformations or prematurity are quite exclusive, and parents’ dependency on specialised personnel is obvious. Therefore, we cannot overlook the fact that a dependency bias might be present in our study, which could appear in parents’ reported healthcare satisfaction. It is also possible that the high healthcare satisfaction might be related to the instrument used (PedsQL), which refers mainly to satisfaction with the in-hospital healthcare. For healthcare satisfaction at home, and to clarify satisfaction with eHealth in care, the question would have needed a different approach. This can be subjected in questionnaire validations for eHealth in future.

### Strengths and limitations

The strengths of this study are that participating parents were represented by both mothers and fathers, and that two of the three questionnaires were formally validated. The drop-out analysis showed that parents not answering the questionnaires completely or at all, used the eHealth device to a lesser extent than parents who had answered the questionnaires completely and were included. The results of the drop-out analyses reflect that an unconsciousness selection bias might be evident, i.e., the study group was constituted by parents who had a more positive attitude to the use of eHealth and therefore answered the questionnaires more completely. This information is important and indicates that there is a group of parents that need attention if they are not using eHealth communication as expected. These parents could be offered more telephone or physical counselling as replacement for eHealth solutions.

The limitations of this study include a low response rate, possibly implying that responders did not necessarily represent the eligible population. As the number of participants became limited, the possibility to find significant results on possible associations were reduced. Also, the very limited distribution of degree of eHealth literacy among participants could have influenced the possibility to statistically identify associations with usage. The limited study time of 30 days can be considered to influence the magnitude of assessment. This cut-off was chosen only in order to study the first time at home after discharge from hospital. This time has been shown to constitute the most fragile time for parents, regarding both the child’s physical complications and psychological demands on families. Since parents were allowed to keep the device longer, a prolonged usage of eHealth is undergoing further analyses within in adapted study settings. Another limitation was that all parents included were required to be able to read and write Swedish, since the study questionnaires were only provided in Swedish. In daily clinical practice, parents with other primary or native languages are also offered the eHealth device.

Lastly, the question regarding satisfaction of the eHealth device was not validated. Since the concept of technology in healthcare is a combination of physical products, processes, and services of care [31], parents might have not reported on the same parts of the eHealth intervention, but instead, for example, about the application itself, the care service connected to it, or the access to highly specialised care. Considering this, they may not have reported on their satisfaction with the specific device itself, although this was specifically requested. Reasons for satisfaction/dissatisfaction with the specific eHealth device and the whole concept around it, needs further investigation. This is also important regarding the ongoing extended use of eHealth and the eHealth device, such as in pediatric nephrology, pediatric cardiology and pediatric oncology – areas in which eHealth and the eHealth device are being investigated within our eHealth research project. Even greater amounts of data and case-control results are therefore to be expected, and strategies are aimed at designing personalized adaptations of eHealth. Evaluations, according to the eChildHealth’s research protocol [32], include outcomes such as end-user experiences, medical safety, psychological effects, and cost-effectiveness.

## Conclusion

In this study, usage of eHealth in transition to home after discharge from advanced paediatric in-hospital care, was associated with parental satisfaction with the eHealth device but not with eHealth literacy or with healthcare satisfaction. To provide safe and equal usage of eHealth, the user-friendliness of the eHealth device seems to be crucial. To improve use of the eHealth device, a proper introduction to it should be offered, ensuring that all parents are able to use it before leaving hospital, and that if technical problems occur, an immediate exchange of non-working equipment is facilitated.

### Electronic supplementary material

Below is the link to the electronic supplementary material.

Supplementary Material 1

## Data Availability

The data set generated and analysed during the current study cannot be shared in order to maintain participants’ anonymity and confidentiality but are available from the corresponding author on reasonable request.

## List of abbreviations

eHLQ: eHealth Literacy Questionnaire
PedsQL: Paediatric Quality of Life
