# Supplementary material for: eHealth usage among parents to premature or surgically treated neonates: associations with eHealth literacy, healthcare satisfaction or satisfaction with an eHealth device
Source: BMC Pediatr. 2023 Oct 21;23:524. doi: 10.1186/s12887-023-04340-3 (PMC10589995; doi:10.1186/s12887-023-04340-3)
Supplement: Supplementary file 1 — Supplementary Material 1 [file 12887_2023_4340_MOESM1_ESM.docx]

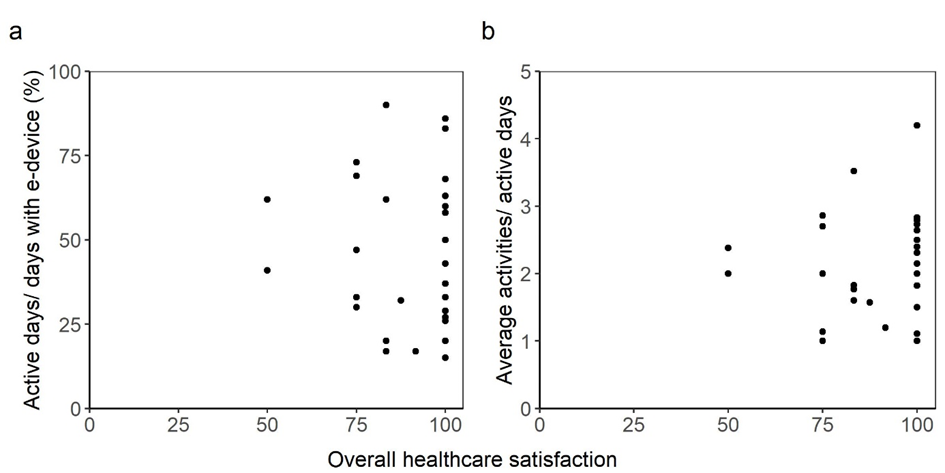


**Supplemental Figure 1a and b.** Association between healthcare satisfaction and eHealth usage.

Scatter plots on overall healthcare satisfaction (dimension 6 in PedsQL) versus eHealth usage, a) active days (%) and b) average activities. No visible linear relationship could be seen, which was also confirmed by the linear regression analysis p=0.540 and p=0.833, respectively.
